# Molecular biology and integrated strategies for activating cryptic biosynthetic gene clusters toward next-generation antibiotic discovery

**DOI:** 10.3389/fbinf.2026.1893206

**Published:** 2026-08-25

**Authors:** Veilumuthu Pattapulavar, Sathiyabama Ramanujam, Avanthi Yellampalli, Henu Sree Badiginchala, John Godwin Christopher

**Affiliations:** 1 Department of BioMedical Sciences, School of BioSciences and Technology, Vellore Institute of Technology (VIT), Vellore, Tamil Nadu, India; 2 Department of Physics, School of Physics, Madurai Kamaraj University, Madurai, Tamil Nadu, India; 3 Department of Integrated Biotechnology, School of BioSciences and Technology, Vellore Institute of Technology, Vellore, Tamil Nadu, India

**Keywords:** antimicrobial resistance (AMR), CRISPR-Cas activation, cryptic biosynthetic gene clusters (BGCs), eco-evolutionary insights, genome mining, heterologous expression, machine learning in natural product discovery, metabolomics

## Abstract

Antimicrobial resistance (AMR) has been identified as one of the 21st century’s severest global public health crises. AMR led to an estimated 4.95 million deaths in 2019 and will claim 10 million lives a year by 2050 in the absence of targeted interventions. During the same period, the number of novel antibiotics discovered has decreased drastically as many researchers are rediscovering known antibiotics, non-model microorganisms are poorly understood or difficult to culture and antibiotic research and development investment has declined drastically. However, high-throughput whole genome sequencing and the subsequent application of bioinformatics in bacterial and fungal genomes have shown that a numerous of cryptic or silent biosynthetic gene clusters (BGCs) remain latent at ambient laboratory conditions since their genes are transcriptionally inactive. Cryptic BGCs represent a vast source of unique secondary metabolites, many of which may yield novel antibacterial, antifungal, anti-cancer and other potentially valuable natural products. This review discusses the biological relevance of cryptic BGCs, the major limiting factors that restricts their activation and novel strategies that have been employed to activate them and exploit their potential to produce novel natural products. The review focuses on biological approaches including CRISPR-Cas mediation for the activation of cryptic BGCs, promoter engineering, pathway refactoring, and heterologous expression; biochemical strategies such as Osman, OsMAC, Precursor Feeding, Chemical Elicitation, Epigenetic Regulation and Co-cultivation and technology-based strategies such as Genome mining, Microfluidic Cultivation systems, High-Throughput Screening, Metabolomics, Molecular Networking and Artificial Intelligence and Machine Learning based prediction of BGCs and their metabolites. The use of multi-omics technologies combined with synthetic biology to achieve better discovery, characterization and large-scale production of novel natural products is also discussed herein. Finally, we will talk about the ecological significance and evolutionary advantage of cryptic BGCs’ role in interactions between microorganisms, such as competition, communication, symbiosis and environmental adaptability, so as to provide a useful background for accelerating next-generation antibiotics.

## 1 Introduction

Rising antimicrobial resistance (AMR) has become a global public health concern in the twenty-first century. Pathogens that formerly responded to antibiotics have become resistant to these drugs, hence rendering many first-line treatments ineffective. With the potential to cause 10 million deaths annually (Nazir, et al., 2025). This disaster underscores a pressing need to discover antibiotics with new mechanism of action and to diversify the natural product discovery panorama, during the “golden age” of antibiotic discovery (1940s–1960s), most clinically relevant antibiotics had been derived from microbial natural products, especially from *Streptomyces* and different actinomycetes. However, the pace of discovery declined substantially after the 1980s because of the rediscovery of regarded compounds, diminishing financial incentives, and demanding situations in cultivating unexplored microorganisms (Olga, 2017; Lewis, 2020). Although development of new antimicrobials is critical, the number of candidates in the antibiotic pipeline is quite restrictive given the scientific, economic, and regulatory issues. Currently, sequencing bacterial and fungal genomes has generated vast libraries of cryptic, so called silent biosynthetic gene clusters (BGCs) whose expressed products have not yet been characterized since they are unable to be produced during normal laboratory cultivation (Medema et al., 2015; Hoshino et al., 2019). The WHO’s 2021 document “Antibacterial marketers in scientific and Preclinical improvement” highlighted that of extra than forty-three antibacterial marketers within the clinical pipeline, only a handful represented simply novel chemical classes indicating a profound innovation gap in antibiotic development (World Health Organisation, 2021). The few agents in preclinical stages by and large target Gram-positive pathogens, leaving Gram-negative infections increasingly untreatable. With the advances in the whole-genome sequencing, it has been easy to discover a number of previously unknown biosynthetic gene clusters (BGCs), some of which are anticipated to synthesize secondary metabolites such as antibiotics, pigments, siderophores, and immune suppressants (Rutledge and Challis, 2015; Ziemert et al., 2016). An immense potential resource of undiscovered molecules remains dormant under conditions used in the majority of studies in laboratories, due to an abundance of dormant gene clusters (Cimermancic et al., 2014). Various genome mining toolkits (antiSMASH, PRISM, and DeepBGC) have been developed, allowing systematic identification of and a prioritized approach toward experimental activation of these cryptic gene clusters (Blin et al., 2021). The important challenge now lies in activating these cryptic BGCs and linking them to their chemical outputs. Many continue to be silent due to tight transcriptional law, loss of environmental stimuli, or dependence on unknown regulatory proteins. The process of activating silent biosynthetic gene clusters through metabolic modification is known as the reprogramming of cellular metabolism in order to activate biosynthetic pathways that have never been used or activated before in standard laboratory growth conditions. However, this strategy is more than just genome editing and encompasses the implementation of several approaches that can modify genetic, regulatory, and physiological elements controlling secondary metabolite synthesis, including altering promoters, redesigning metabolic pathways, varying the number of transcription factors, expression in heterologous hosts, the CRISPR-Cas approach for genome editing, modification of regulation through synthetic biology approaches, as well as the implementation of auxiliary strategies such as the cultivation of mixed microbial cultures, epigenetic modifications, and environmental stressors. These synergistic, counterbalancing approaches circumvent natural regulatory constraints on expression, recruit and activate silent pathways, and enable a *de novo* exploration of uncharted secondary metabolite chemical space. (Rutledge and Challis, 2015). Modern approaches including heterologous expression, promoter refactoring, co-cultivation, and CRISPR-Cas based activation are an increasing being used to overcome those obstacles (Zhang et al., 2019; Hoshino et al., 2019). Demonstrated that co-culturing *Streptomyces* species with my colic acid containing microorganism can successfully activate silent biosynthetic pathways, leading to the production of new secondary metabolites otherwise undetectable in monoculture. Such ecological simulation and chemical communique strategies mirror how microbial interactions in herbal environments power secondary metabolism. Furthermore, the combination of artificial biology, systems biology, and machine learning is revolutionizing natural product discovery. By way of computationally predicting enzyme functions and molecular scaffolds, researchers can now prioritize promising BGCs for experimental activation. Genome-editing tools like CRISPR-Cas9 and excessive-efficiency homologous recombination in hosts which include *Streptomyces*, *E. coli*, and *Saccharomyces cerevisiae* allow for targeted activation and combinatorial biosynthesis of novel molecules (Smanski et al., 2016). From an eco-evolutionary perspective, cryptic BGCs are not merely dormant but may additionally represent adaptive responses to environmental pressures which includes nutrient limitation, interspecies competition, or host protection (van der Meij et al., 2017). The study of natural environmental activation stimuli might help us know a lot about the process of activating silenced biosynthetic gene clusters. Consequently, reprogramming microbial metabolism *via* multi-omics, CRISPR primarily based activation, and ecological mimicry signals a paradigm shift from random screening to rational, programmable discovery. Harnessing the untapped potential of cryptic BGCS could usher in a new era of the antibiotic discovery. By viewing microorganisms as programmable chemical factories, different methodologies such as metabolic reprogramming, synthetic biology, and genome engineering can turn microbial cells into sustainable biofactories for developing next generations of antibiotics. In addition, the fast development of the genome sequencing techniques has made the process of identifying biosynthetic gene clusters (BGCs) much quicker. The first complete bacterial genome, belonging to *Haemophilus influenza*, was published in 1995, and since then and thanks to advancements in next-gene sequencing technology, sequencing capacity has gained in efficiency and price availability. (Fleischmann et al., 1995), reaching over 700 in 2012 alone (Bertelli and Greub, 2013). Increased throughput, enhanced fidelity, and reduced costs related to next-generation sequencing technologies have all contributed to this quick growth of genomic information (Shendure and Ji, 2008; Bertelli and Greub, 2013). Afterwards, projects to sequence genomes have developed from targets among disease-causing microbes to various types of microbes of importance from industrial or environmental point of view, thus unveiling the exceptional capacity for biosynthesis of actinobacteria and especially of *Streptomyces* as the leading producers of a vast variety of antibiotics and other bioactive secondary metabolites (Challis and Hopwood, 2003; Nett et al., 2009) After that the developments led to the emergence of the generation technique called genome-guided natural product discovery which has allowed the systematic identification of the inactive biosynthetic gene clusters.

## 2 BGC cloning and assembly: heterologous expression of fungal BGCs in *Saccharomyces cerevisiae*

Fungal BGCs normally have around 10 genes encompassing core biosynthetic gene enzymes (BG enzyme genes), tailoring gene enzyme genes (TGC genes), transport genes (TT genes) and self-resistance gene enzymes (SR genes) (Mattern et al., 2015). Considering the need to transfer many genes and the vast dimensions of certain cores, one of the many biosynthetic enzymes (PKS, NRPS, and hybrid enzymes). Fungal BGCs are challenging to express in a heterologous system, especially those BGCs bearing large clusters, such as PKSs (polyketide synthases), NRPSs (non-ribosomal peptide synthetizes), and hybrid PKS-NRPS. Efficient strategies, including new cloning and assembling procedures using *S. cerevisiae*, a major host in heterologous expression with widely exploited genetics tools and excellent homologous recombination power, as we proposed with those approaches here with BGCs expressed in either a chromosome-integrated pathway or Episomal plasmids, which facilitate chromosomal integration for genes to be stably expressed after many generations under re-sub cultivation and Episomal expression controlled by varying the copy number for gene dosage adjustments. Meanwhile, the CRISPR-Cas9 genome editing method contributes remarkably high efficiency for specific multi-locus gene integration as well as for constructing gene clusters. The high-throughput homology recombination and modern genome editing tools allow efficient cloning, assembling, and heterologous expression of fungal BGCs, including the large clusters, leading to efficient one-step assembly and expediting new natural product isolation. (Jakočiunas et al., 2015; Meng et al., 2022). A summary of the key features, challenges, and strategies involved in the heterologous expression of fungal BGCs in *Saccharomyces cerevisiae* is presented in Table 1.

**TABLE 1:** Heterologous expression of fungal BGCs in *Saccharomyces cerevisiae*.

S.no	Aspect	Details	References(s)
1	Average number of genes in fungal BGCs	Approximately 10 genes	(Keller, 2019)
2	Gene components	Core biosynthetic genes (PKS, NRPS), tailoring enzymes, transporters, and self-resistance genes	(Brakhage and Schroeckh, 2011)
3	Challenges in transferring large BGCs	The biggest obstacle of studying a large biosynthetic gene clusters, which is comprised of the genes from PKS and NRPS families, is their complex size and other difficult factors	(Luo et al., 2019)
4	Preferred host	*Saccharomyces cerevisiae* (established genetic tools and efficient homologous recombination).	(Luo et al., 2019)
5	Expression strategies	- Genome integration - Episomal plasmids	(Valette et al., 2017)
6	Advantages of Episomal plasmids	Adjustable gene dosage *via* plasmid copy number	(Ro et al., 2006)
7	CRISPR-Cas9 utility	Site-directed multipanel gene integration and highly efficient genome engineering	(Jakočiunas et al., 2015)
8	Homologous recombination	Highly functional in yeast and enables a single step assembly of large BGCs and its concomitant integration into the yeast genome.	(Valette et al., 2017)
9	Genetic stability	Gene amplification and chromosomal insertion result in consistent expression over multiple subcultures.	(Harvey et al., 2018)
10	Latest achievements	Fungal BGCs have been successfully assembled in a one-step manner.	(Liu et al., 2019; Cai et al., 2022)

## 3 Assembly and BGC cloning for heterologous bacterial BGC expression in *Saccharomyces cerevisiae*

### 3.1 Expansion of bacterial genome mining and its impact on BGC discovery

Genomics and Natural Product Discovery Bacterial genome sequencing is quickly growing to yield a wealth of new BGCs that previously went unrecognized. The first complete sequence of a bacterial genome was achieved in 1995 for *Haemophilus influenza*, but after subsequent developments in new-generation sequencing (NGS) methods dramatically decreased price, growing throughout and precision concurrently, greater than 1,000’s of full-length bacterial genomes have been deposited in archives each year, now giving us the power to genotype-mine on an even better degree (Fleischmann et al., 1995; Medema et al., 2015). Genome studies have revealed that up to 40 BGCs may be carried per strain for some bacteria, the majority of these being in a transcriptionally inert state during normal growth-room and laboratory conditions of *in vitro* cultures. This resulted in a revolution in antibiotic discovery from traditional bioactive-based screenings towards genome-based methods that target, activate and rank encrypted BGCs for *in vitro* production using *in vitro* gene activation strategies or *via* synthetic biology approaches (Cimermancic et al., 2014).

### 3.2 Limitations of native bacterial hosts for BGC expression

Native bacterial hosts, including well known *Streptomyces* and other actinomycetes, show huge capacity in natural product biosynthesis, however these may cause limitations during functional characterization of BGCs. These include slow growth rates, distinct developmental stages and processes, difficult or inefficient genetic manipulation (e.g., Transformation), highly co-regulated biosynthetic gene expression systems which do not readily function in a laboratory setting (Baltz, 2017). This means that they are best transferred to heterologous hosts for expression. *Saccharomyces cerevisiae* has proved a very successful, genetically easy-to-work-with chassis to be used as a source of tools for the cloning, engineering, and functional analysis of bacteria’s biosynthetic pathways under minimal native regulation (Editorial: Yeast synthetic biology: new tools to unlock cellular function, 2015).

### 3.3 *Saccharomyces cerevisiae* as a host for bacterial BGC assembly

*Saccharomyces cerevisiae* is a favourable yeast chassis for cloning and assembly of large bacterial BGCs because of its efficient homologous recombination machinery. TAR-cloning (transformation-associated recombination cloning) could also be used to capture directly and assemble BGCs larger than 100 kb, indicating that the yeast is excellent at designing large PKS and NRPS pathways (Kouprina and Larionov, 2006). While *S. Cerevisiae* often serves as a heterologous expression host for fungal BGCs, one of the main applications of yeast in bacterial BGC study is high-throughput cloning, assembly, and bioengineering of large biosynthetic pathways prior to the introduction into bacterial hosts. It can accelerate the identification and characterization of new bacterial natural products through promoter swapping, Pathway Refactoring, gene Dosage Optimization, modular assembly, *etc.* (Lee et al., 2015; Smanski et al., 2016).

## 4 Integrated strategies for activating cryptic biosynthetic gene clusters in antibiotic discovery

Under normal laboratory conditions the genomes involved in the synthesis of characteristic secondary metabolites with antibacterial functions in bacteria are transcriptionally dormant and inaccessible to genetic manipulation. Therefore, these are often referred to as “cryptic” biosynthetic gene clusters (BGCs), being abundant in the *Streptomyces* and in other well-known producers of secondary products. It has been known for several years, from sequencing studies, that one organism alone contains 20 to 40 BGCs; of these, only a fraction will be expressed in laboratory cultures under normal conditions (Rutledge and Challis, 2015). The BGC reservoir identified is promising for a future source to identify new antibiotics targeting resistance. For the activation of BGCs which remain silent the combination of diverse strategies is needed, broadly categorized as (i) molecular biology and genetics-based approaches comprising heterologous gene expression, promoter engineering, CRISPR-Cas mediated genomic modification, and pathways refactoring; (ii) Biochemical methods that include the so-called One Strain Many Compounds (OSMAC) approach, precursor feeding, chemical elicitation, and epigenetic modification; and (iii) technology or cultivation-based strategies that involve the co-cultivation of strains, use of microfluidic cultivation devices, or high-throughput screening methods. The synergistic application of those methods allowed to substantially increase the yield of activated BGCs, and accordingly resulted in an accelerated discovery of new biologically active secondary products (Ziemert et al., 2016; Blin et al., 2021). Alternatively, the process has become more accessible thanks to the advent of genome mining tools like antiSMASH and PRISM that allow one to efficiently predict, identify, and rank any cryptic BGC using conserved motifs and domain architecture (Ziemert et al., 2016; Zhang et al., 2019).

### 4.1 Molecular biology activation strategies

Genome sequencing reveals that there is a lot of latent chemical space contained within bacteria - particularly in *Streptomyces* and related actinomycetes, as well as some filamentous fungi. In bacteria, we identify dormant gene clusters called ‘biosynthetic gene clusters’ (BGCs) that are silent under standard conditions but encode for novel secondary metabolites with the potential for antibacterial, antifungal or anti-cancer properties. Most of these chemical products have never been seen before (Pillay et al., 2022; Wang and Mao, 2025). Organic compounds produced by genomic regions, also known as orphaned or unidentified biosynthetic gene clusters (BGCs), lack natural metabolites. Although these clusters cannot be connected to any noticeable substance under usual conditions, genome sequencing is often used to locate them because of their silent or invisible appearance. Estimates indicate more than 90% of the microbial genome BGCs encode for secondary metabolites either unknown or uncharacterized (Cimermancic et al., 2014; Medema et al., 2015). Most microbial BGCs are accounted for by this group. Symbolizing this untapped store of chemical creativity, these unknown BGCs may harbour new structural classes of antibacterial, antifungal, or anticancer agents. But it is hard to link these clusters to their metabolites since there are regulatory issues, gene silencing, or a lack of knowledge of their biosynthetic origin. Metabolomics, synthetic biology, and bioinformatics (such anti SMASH and Deep BGC) advancements are helping to close this divide. Through molecular networking, the mix of genomic mining and mass spectrometry-based metabolomics, for instance, enables linking of gene clusters to distinctive compounds (Casanovas et al., 2015). Give experiments top priority, machine learning techniques are also being used to predict the function of unknown BGCs depending on sequence characteristics (Hannigan et al., 2019). Unlocking these unexplored BGCs is also crucial for improving the chemical variety of known natural products in addition to satisfying the critical demand for new antibiotics in the face of rising drug resistance.

### 4.2 Biochemical approaches for activating cryptic biosynthetic gene clusters

An additional and very powerful technique to trigger silent BGCs does not involve genetic modification of the producing microorganism, but modulation of microbial physiology and conditions. This kind of method is referred as a bio-synthetic activation or a culture-based approach since it uses alterations of medium components, chemical signals or cellular conditions to activate biosynthesis rather than direct molecular manipulation. One widely applied bio-synthetic activation method in microbial natural products research is the One Strain-Many Compounds (OSMAC) strategy. Through variations in parameters of cultivation, for example, sources of carbon or nitrogen, pH value, temperature, concentration of oxygen or salt, incubation time, or chemical stimulators, one strain can be induced to generate diverse secondary metabolites under laboratory conditions, the occurrence of which may not be seen in normal standard laboratory cultivation (Bode et al., 2002; Rutledge and Challis, 2015). Another widely applied biochemical strategy, Precursor feeding is to introduce intermediate biosynthetic molecules to microbial cultures in an attempt to divert metabolome flux toward overproducing secondary metabolites of the target compound. Similar to that strategy, chemical elicitors such as N-acetyl glucosamine -butyrolactones and rare earth elements serve as signalling molecule to trigger specific transcription regulators of path-ways, eventually leading to induction of dormant BGCs. In addition, in filamentous fungi, epigenetic approaches have successfully used for inducing dormant clusters through application of DNA methyltransferase (DNMT) inhibitors and histone deacetylase (HDAC) inhibitors to change chromatin structure to be accessible to the repressed clusters (Bertrand et al., 2014). These biochemistry-based approaches complement genetic based approaches including the tools available in both molecular biology and synthetic biology in that they can recapitulate conditions found during microbial life in either its natural ecological or physiological environments. In conjunction with approaches like genome mining, metabolomics, and heterologous expression, biochemical stimulation expands significantly the capabilities for isolating new structures to aid the search for and discovery of new potential novel antibiotic scaffolds against resistant pathogens (Lewis, 2020).

### 4.3 Eco-evolutionary significance of cryptic biosynthetic gene clusters

In particular, it is hypothesized that they are responsible for the production of secondary metabolites, which have a large impact on the interactions between microorganisms (such as conflict, symbiosis, and communication) and help them adapt to their local environment. The study of such gene clusters has large implications for our understanding of microbe ecology and evolution and natural regulation (Ji et al., 2024). Microorganisms have evolutionarily diversified into making thousands of types of secondary metabolites, which serve both as offensive chemical warfare and signalling molecules that allow them to compete, socialize, and exist in complex ecological settings. For this reason, many bacterial secondary metabolite biosynthetic genes are passed along from one generation to another simply because having them affords them advantages in combatting competitors, influencing host-microbe relationships, and tolerating extreme conditions (Davies and Davies, 2010). Evolutionary perspective: the majority of our BGCs are currently silent in the lab because gene cluster expression is frequently controlled by a specific environmental cue like nutrient starvation, interaction with other competitors, host cue signals, or stress responses of the ecosystem (Zhang et al., 2019). The natural functionality of these clusters is seemingly strongly related to adaptation to the environment as opposed to the constitution of a metabolite. Clarifying the eco-evolutionary role of cryptic biosynthetic BGCs has potential impact beyond a pursuit of new antibiotics; it also contributes knowledge about how microbial diversity evolved through chemical diversity itself. From an ecological perspective, these metabolites may affect many aspects of the community dynamics of micro-organisms, affect how communities respond to environmental change, or aid in the maintenance of community health and resilience (Van der Meij et al., 2017).

## 5 Challenges in activating cryptic biosynthetic gene clusters (BGCs)

### 5.1 Transcriptional silencing of cryptic BGCs

Cryptic BGCs are frequently silent due to the loss of their native regulatory networks which are inactive or only respond to limited specific environmental stimuli that do not occur during common laboratory cultivation (Wang and Mao, 2025), Production of little or no metabolite consequently occurs and results in poor characterization of functionality. This silence is common with regulated promoters that respond only in specific conditions, the absence of appropriate stimuli in laboratory settings and by the expression of either global or cluster specific regulatory protein repressors (Laureti et al., 2011) In natural environment these regulatory networks are activated by the presence of other microorganisms (nutrient limitation, competition, *etc.*) and signal delivered by a host, however in laboratory production it will be hard to provide this exact environmental conditions (Rutledge and Challis, 2015). Furthermore, many cryptic BGCs reside inside of regulator-composed network where include positive and negative regulators, repressor and feedback system, which making it is really tough to activate without influence on the hosts themselves. Therefore, cracking transcriptionally silencing barrio provides opportunity for discovering novel natural products.

### 5.2 Unknown activators and complex regulatory networks

The intricate control and unknown activator expression depend on transcription factors, sigma factors, and feedback loops. Recreating them *in vitro* is difficult since the activating stimuli or events are often unknown (Pillay et al., 2022; Ji et al., 2024). In the BGC context, the expression of numerous cryptic BGCs requires interactions of transcriptional and sigma regulators, feedback control and cluster located regulators (CSRs) in what is often a highly organized regulatory network. Reproducing the *in vivo* regulatory environment *in vitro* is challenging as the inductive conditions remain often elusive or Unknown the physiological environmental trigger of regulation is Unknown. Like quorum sensing molecules, nutrient availability, or host-specific metabolites not found in laboratory culture environments, these activators could be sensitive to certain chemical or environmental stimuli (Medema et al., 2014). Though possessing the genetic ability to create bioactive chemicals if these stimuli are not found, the path remains silent. Furthermore, controlled by hierarchical regulatory networks, enigmatic BGCs include CSRs interacting with worldwide regulators to coordinate metabolic state, developmental phases, and stress reactions (van der Meij et al., 2017). Feedback inhibition, crosstalk with unrelated pathways, or conditional activation only during specific developmental stages or in the presence of competing organisms are examples of the control (Traxler et al., 2013). As an environmental defense mechanism, this complex control evolved to stop unnecessary metabolite production in energy-expensive pathways unless it is crucial for survival. Consequently, finding these BGCs calls for regulatory gene identification, synthetic activator development, and ecological mimicking methods simulating the natural triggers for route activation.

### 5.3 Incomplete functional annotation of BGC genes

Poor functional annotation because many of the genes in cryptic BGCs encode for hypothetical or unknown proteins (Liu et al., 2021). Research on buried biosynthetic gene clusters (BGCs) is hampered by the absence of appropriate functional identification of these genes, which renders forecasting the chemical compounds and their bioactivities challenging. Many of these are still just highly annotated without any experimental basis, such as “putative polypeptide synthase” or “no ribosomal peptide synthetize” (Medema et al., 2014). Genome sequencing unveiled an astonishing richness in predicted BGCs; however, their functional characterization remains difficult, as most contain newly discovered enzymes having unknown functions and displaying similarity with existing sequences with only few or no homologous ones available in known databases (Cimermancic et al., 2014), Lack of complete functional annotation means that prioritized experimental validation and prediction of scaffolds derived from BGC activation are not possible However a number of breakthroughs have recently been reported which are beginning to improve functional prediction from computational analysis approaches—via machine learning (ML)-based tools such as PRISM, DeepBGC, – or *via* structure-based bioinformatics approaches which allow relating sequence to function (Hannigan et al., 2019), However, functional annotation needs to be refined to access capabilities of cryptic BGCs and is still improvable.

### 5.4 Instability and metabolic burden

It is challenging to forecast metabolic logic or metabolite structures. When artificially activated, some BGCs put a major metabolic burden on the host leading to toxicity, poor yields, or instability (Bian, 2025). A crucial issue when attempting to utilize cryptic biosynthetic gene clusters is that they are genetically unstable, imposing a metabolic challenge upon the host organism in the process. Several BGCs are found in genomic islands or plasmids that, under stress or during laboratory maintenance, are susceptible to recombination, deletion, or rearrangements (Bentley et al., 2002). Thus, competition for endogenous resources, reduction in growth rate, low viability, and little output are usually observed when pathways are introduced in foreign hosts that are not intrinsically attuned to their biosynthetic demands (Komatsu et al., 2010). Microorganisms often fine-tune BGC activation to avoid unnecessary metabolic stress in response to nature-related environmental signals. Still, in the laboratories, constitutive expression or promoter-driving ensured expression may imbalance the host metabolism. Mitigating methods involve precursor supplementation; balancing it through expression tuning; dynamic control systems; and resorting to sturdy chassis organisms such as *Streptomyces* or genetically engineered strains of *E. coli* for secondary metabolite production (Smanski et al., 2016). Thus, the issues of metabolic burden and genetic stability would need to be addressed in turning cryptic BGC activation into a reliable and scalable antibiotic discovery pipeline.

### 5.5 Cloning and expression difficulties

Large or high-G + C cryptic BGCs are technically problematic for their cloning and heterologous expression and frequently need substantial pathway modification and judicious selection of heterologous expression hosts (Ji et al., 2024). Using cryptic biosynthetic gene clusters (BGCs) is always hampered by the complexity of cloning and getting functioning expression of large, intricate gene clusters in suitable host systems. Many BGCs, often spanning tens to hundreds of kilobases, include several operons, regulatory elements, and strange GC-rich sequences difficult to clone by standard molecular biology methods (Zerikly and Challis, 2009). Furthermore, certain biosynthetic enzymes like such big polypeptide synthases (PKSs) or no ribosomal peptide synthetises (NRPSs) are well known to be challenging to express in soluble and active forms even in their original producers (Komatsu et al., 2010). Their size, complexity of folding, and need of specific chaperones account for this.

**FIGURE 1:**
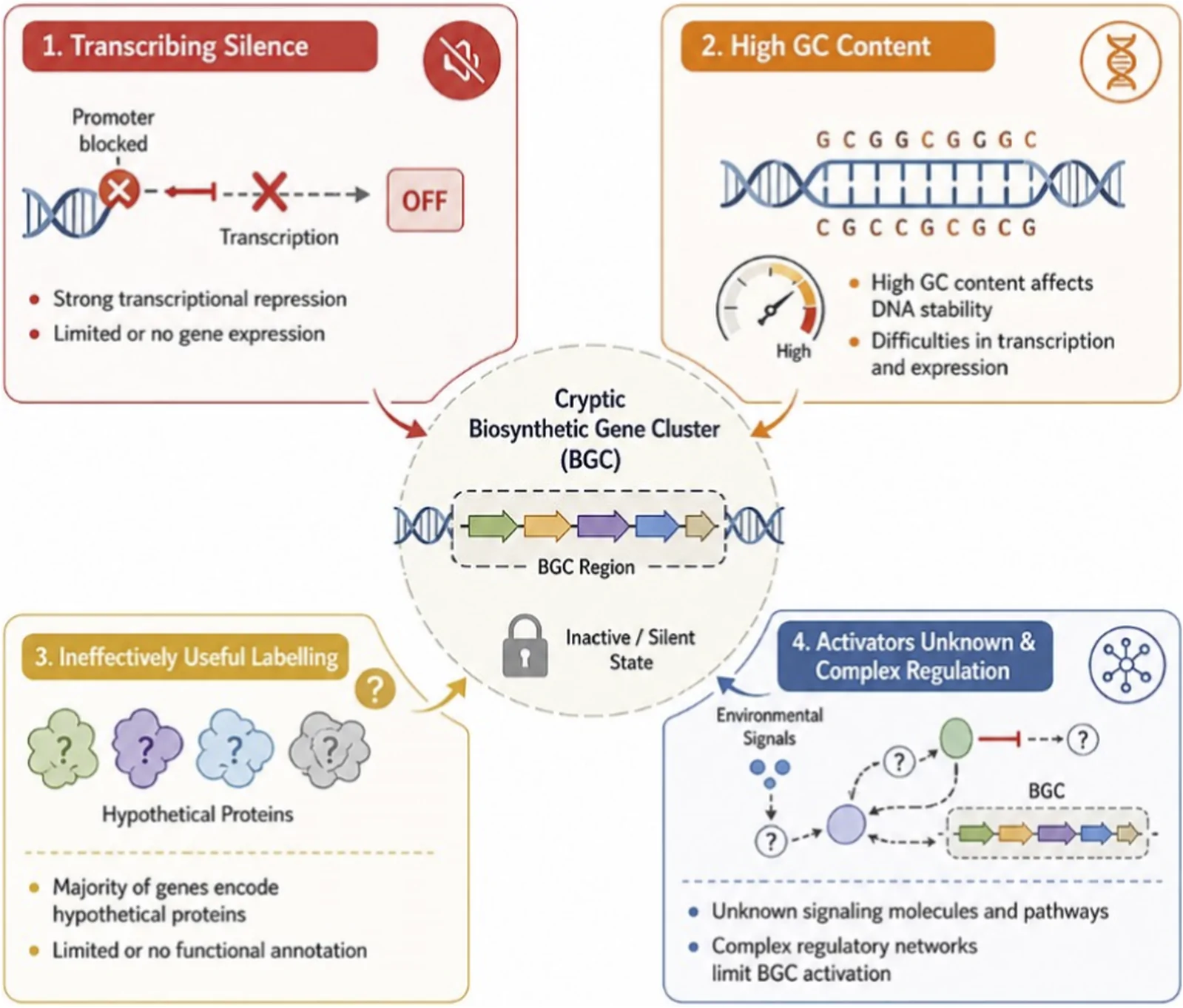
Major hurdles to activate and exploit cryptic biosynthetic gene clusters (BGCs) of genes.

The major obstacles that restrict the activation and functional exploration of cryptic BGCs, including transcriptional silencing, GC-content-related cloning difficulties, incomplete functional annotation, and complex regulatory networks, are summarized in Figure 1. The illustrated figure recapitulates the key obstacles that restrict the activation and functional exploration of cryptic BGCs in microbes, such as transcriptional silencing, low or high GC content, lack of functional annotation of BGC genes, complex regulatory network (left). These impediments in identifying novel bioactive compounds hinder mining cryptic BGCs that, along with the genome, the combination of biology, multi-omics and genome-mining strategies is a powerful solution (right).

## 6 Cryptic BGCs are hidden resource deposits

Biosynthetic gene clusters (BGCs) are groups of associated genes that encode the enzymes and regulatory proteins required for synthesis of specialized metabolites, including antibiotics, pigments or siderophores in microorganisms. Many genome sequences have revealed multiple BGCs within microorganisms that are cryptic or silent, which means that they are not expressed under standard lab culture conditions, or their precursors or products cannot be detected (Wang and Mao, 2025). Invisible metabolic elements include a plethora of chemical diversity that we previously have not examined, especially in unexplored environments or uncultured microbes, and offer potential access to new sources for antibiotic discovery (Zhang et al., 2023). A major source of new medications is natural materials (Newman and Cragg, 2020). Homologous expression of the targeted biosynthetic gene cluster (BGC) in native species using genome editing and heterologous expression of the BGC in suitable hosts following its cloning and modification in model bacteria or yeast (Bachmann et al., 2014; Huo et al., 2019) are the main approaches for genome mining. Still, the classical OSMAC (one strain, many compounds) method and genomic mining approaches have not been able to meet the ongoing demand for scalable identification of novel compounds despite 3 decades of thorough genomic mining of bacterial natural products (Scherlach and Hertweck, 2021). The genus *Streptomyces* is noted for producing a great diversity of bioactive natural chemicals and prospective medicines (Atanasov et al., 2021). But the search for previously unknown natural products from *Streptomyces* species is hampered by the labour intensity or lack of highly effective gene editing techniques for turning on hidden BGCs in indigenous strains. Focusing on accomplishing the scalable genome mining of “unseen” *Streptomyces* natural products in an autologous manner (Xie et al., 2024), recently published their ground-breaking work developing a CRISPR-Cas9-based ACTIMOT (Advanced Cas9-mediaTed *In vivo* Mobilization and Multiplication of BGCs) system whereby the spreading process of antibiotic resistance genes (ARGs) has been artificially simulated to mobilize and amplify extensive genomic BGCs (Bian, 2025).

## 7 Future directions - towards a new era of antibiotics

The strong call for new antibiotics because of the growing danger that antimicrobial resistance is going to overwhelm the current antibiotic reservoirs is the major reason to go back to the drawing board on discovery pipelines and scrape the surface of the cryptic and uncharacterized biosynthetic gene clusters (BGCs) like never before. An integrative approach combining bioinformatics, synthetic biology, systems biology, and machine learning will be required to break through the hurdles faced by current methodologies. Not only will genome mining tools be able to find BGCs, but they will also be able to anticipate their chemical outputs with higher accuracy by employing deep learning algorithms and structural prediction tools (Skinnider et al., 2017; Hannigan et al., 2019). Synthetic biology concurrently is progressing towards achieving the targeted BGCs refactoring, modular pathway design, and the fabrication of chassis organisms with suitable heterologous expression that are even capable of high performance (Smanski et al., 2016). Besides that, CRISPR-based regulatory tools can give full-range control of the silent clusters; thus, researchers will be able to change the expression levels and switch on the previously uncaused pathways (Zhang et al., 2019). Alongside laboratory breakthroughs, ecological and evolutionary perspectives are expected to be a major factor in the hunt for antibiotics. Learning the natural provocateurs and ecological functions of BGCs, for instance, interspecies competition or host-microbe interactions, can breathe life into novel cultivation techniques, which are recreations of nature that consequently unveil new pathways (van der Meij et al., 2017). When paired with high-throughput metabolomics and AI-enabled pattern recognition, such approaches may significantly shorten the time needed to link chemical dark matter with genomic frameworks. It is a multidisciplinary and collaborative method that encompasses academia, industry, and policy that will, in the end, lead to success. Databases of BGCs that are accessible to everyone, tools for genome mining that are open source, and global consortia that focus on innovations in antibiotics will be the key factors that will facilitate the sharing of resources and reduce the occurrence of duplication.

## 8 Conclusion

The growing problem of antimicrobial resistance demands solutions that are out of the usual discovery pipelines. One of the most striking new sources of innovating conceptions for the next-generation of antibiotics is the cryptic and uncharacterized biosynthetic gene clusters (BGCs), which are transcriptional inactivity, regulatory complexity, and technical hurdles that have, in most cases, led to their neglect. Technological breakthroughs in genome mining, synthetic biology, and CRISPR-based regulation are redefining microbial metabolism and converting stealth genomic codes into next-generation drug candidates. At the same time, the use of eco-evolutionary knowledge in conjunction with machine learning, multi-omics, and high-throughput metabolomics is rapidly bridging the distance from sequence to function, thus unravelling the ecological justification for microbial chemical diversity. Nevertheless, the realization of this future extends beyond the scientific innovation alone - it involves the development of collaborative frameworks that encompass academia, industry, and policy for setting up open databases, shared infrastructures, and global networks of innovation. If we no longer view microbes as mere repositories of genetic information but as programmable pharmacies, then we could access the very chemical ingenuity of nature. This transition in thinking from accidental discovery to purposeful, design-driven unmasking of cryptic pathways holds the ability to inaugurate a new epoch of antibiotics, thus, making available sustainable antimicrobial agents in the battle of drug resistance pathogens.
